# Fertility-Associated Polymorphism within Bovine *ITGβ5* and Its Significant Correlations with Ovarian and Luteal Traits

**DOI:** 10.3390/ani11061579

**Published:** 2021-05-28

**Authors:** Jianing Zhao, Jie Li, Fugui Jiang, Enliang Song, Xianyong Lan, Haiyu Zhao

**Affiliations:** 1 School of Life Sciences, Lanzhou University, No. 222 South Tianshui Road, Lanzhou 730000, China; zjn00411@163.com; 2 Laboratory of Animal Genome and Gene Function, College of Animal Science and Technology, Northwest A&F University, Yangling 712100, China; lijie950302@163.com; 3 Shandong Key Laboratory of Animal Disease Control and Breeding, Institute of Animal Science and Veterinary Medicine, Shandong Academy of Agricultural Sciences, Jinan 250100, China; fgjiang2017@163.com (F.J.); enliangs@126.com (E.S.)

**Keywords:** bovine, *ITGβ5* gene, insertion/deletion (indel), ovarian traits, corpus luteum

## Abstract

**Simple Summary:**

The fertility of bovines is essential for cattle husbandry. ITGβ5, which is suggested to be closely related to fertility, is known to mediate cell adhesion and affect a variety of cellular activities. To investigate the relationship between the *ITGβ5* gene and the fertility of bovines, 696 ovarian samples were collected and six potential indel (insertion/deletion) within *ITGβ5* were analysed, from which a deletion mutation was found to be polymorphic. The genotype frequency and allele frequency of this locus in the investigated population were calculated and the population genetic parameters analyzed. In addition, this locus was found to be significantly correlated with ovarian width and corpus luteum diameter. Considering the importance of ovary and corpus luteum in reproduction, it is tempting to speculate the crucial effects of *ITGβ5* on bovine fertility, which still need further validation. The results of our study might provide a theoretical basis for future breeding to enhance bovine reproduction.

**Abstract:**

There is an urgent need to improve bovine fertility, and molecular marker-assisted selection (MAS) can accelerate this process. Genome-wide association studies suggest that *Integrin β5 (ITGβ5)* might affect fertility in bovines. As a member of the integrins family, ITGβ5 can bind to the extracellular matrix and mediate various cellular processes. In our study, primers spanning six potential insertion/deletion (indel) polymorphisms within the *ITGβ5* gene were designed and 696 ovary samples from different individuals, the vast majority not in oestrum were collected for genetic variation detection. A deletion locus, rs522759246, namely P1-D_13-bp_, was found to be polymorphic. The allele D frequency was 0.152 and the polymorphism information content (PIC) value was 0.224, indicating a low-degree PIC. This locus did not follow the Hardy–Weinberg equilibrium (*p* = 1.200E-23). Importantly, associations between P1-D_13-bp_ and ovarian morphological traits were established. Polymorphisms of this locus had significant correlations with ovarian width (*p* = 0.015). The corpus luteum is also linked to fertility and P1-D_13-bp_ was significantly correlated with corpus luteum diameter (*p* = 0.005). In conclusion, an indel mutation within the bovine *ITGβ5* gene was identified, which was significantly associated with several ovarian and luteal traits.

## 1. Introduction

Simultaneous improvement of milk production and fertility is challenging in cattle breeding due to the unfavorable genetic correlation between the milk yield and fertility of cows [1,2,3]. Fertility is a complex trait; several biological pathways are involved in related regulatory processes and fertility levels have low heritability [4]. Compared with traditional breeding, molecular marker-assisted selections (MAS) for fertility-associated traits are more efficient and can speed up breeding [5,6]. Indel (insertion/deletion), which is commonly used in MAS, has the advantages of simple and rapid recognition [7].

Genome-wide association studies (GWAS) are now commonly used to search for candidate genes affecting bovine reproductive performance, providing many sources of reference information. GWAS on non-return rate, fertility treatment, and retention of the placenta have been conducted [8]. The quantitative trait locus for the female fertility index was identified using imputed whole-genome shotgun sequencing [9]. Variants associated with bovine fertility [10], as well as single-nucleotide polymorphism (SNP) markers that are correlated with variation in beef heifer reproduction and performance of their calves, have been identified [11]. Similarly, the association between SNPs within *PRNT* genes and fertility has been explored in goats [12]. However, whether the candidate genes selected through GWAS have practical significance remains to be verified.

Previous GWAS support the integrin family gene *Integrin β5* (*ITGβ5*) as a candidate gene for fertility in bovines [1]. Encoding Integrin β5, *ITGβ5* controls cell adhesion to the extracellular matrix (ECM) and affects cell motility and migration, as well as signaling pathways related to cell growth, differentiation, and apoptosis [13,14]. It is well known that integrin β5 can bind the extracellular matrix and may have a crucial role in ovarian development [15]. Previous studies have suggested that this gene has a significant effect on mouse ovarian development [14], bovine follicle size and bovine follicle growth [16,17], and has an important influence on avian egg laying [18,19]. Given the importance of *ITGβ5*, it is tempting to speculate that the *ITGβ5* gene may be critical in regulating ovarian phenotype and corpus luteum formation in bovines, thus affecting fertility.

As the female gonad, the ovary is a reproductive gland that produces gametes and supplies mainly steroidal hormones [20]. Through its key role in development and egress of ova, the ovary will directly influence ovum production [19]. The corpus luteum (CL), formed by terminal differentiation of granulosa and theca cells, secretes progesterone required to maintain pregnancy. In the absence of pregnancy, it degenerates into corpus albican [21,22]. In addition, the establishment and maintenance of pregnancy involves a complicated interaction between CL, the embryo, and the endometrium [23]. Traits related to ovarian development can evaluate the reproductive performance of bovines. Recently, significant correlations between polymorphisms of *Adenylate cyclase 5* and *hydroxysteroid 17-beta dehydrogenase 3* and traits related to ovarian morphology in cattle were identified, and these correlations have great potential to affect the reproductive performance of bovines, which support the contention that traits associated with ovarian morphology can be used as an effective indicator of female fertility [23,24]. Therefore, in our study, we investigated the polymorphisms of the bovine *ITGβ5* gene as well as their associations with ovarian and luteal traits of Holstein cows.

## 2. Materials and Methods

### 2.1. Collection of Ovarian Tissue Samples from Cows

A total of 696 ovarian samples derived from different individuals were collected from healthy adult Holstein at the slaughterhouse, and 90% of female cows were not in oestrus estimated by types of corpora lutea, follicle and corpus albicans [24]. The D-loop region of mitochondrial DNA was used to verify that all ovaries were derived from different individuals [25].

Morphological phenotypes of these ovarian specimens were determined by the same person with unified criteria, including ovarian length, width, height, and weight, as well as the diameter of the corpus luteum and the number of corpora lutea. In addition, the number of mature follicles was counted and the diameters of the follicles were measured. Ovaries were weighed using a sterile electronic scale and the length, width, height, and diameter of the samples provided were measured using a Vernier caliper or a double ruler.

### 2.2. Extraction of Total DNA

Total genomic DNA was extracted from the collected ovarian tissues according to the salt extraction method reported in the literature [26]. The quality of all extracted DNA samples was assayed by a Thermo NanoDrop 1000 based on the 1.8 < OD 260/280 < 2.0 standard and the integrity was checked by agarose gel electrophoresis. Qualified DNA samples were diluted to a concentration of 10 ng/µL and temporarily stored at 4 °C for downstream experiments.

### 2.3. Selection of Indel Sites and Design of Primers

According to the Ensembl database (http://asia.ensembl.org/index.html) (accessed on 27 May 2021), six indel polymorphisms were selected in the bovine *ITGβ5* gene. The PCR primers for amplification were designed based on the reference sequence of bovine *ITGβ5* and its downstream area (GenBank ID: 282564, NC_037328.1: 69192856-69312146), and were synthesized by Sangon Biotech (Shanghai, China) (Table 1).

### 2.4. Identification of Indel Loci, Genotyping and DNA Sequencing

First, the mixed DNA pools were constructed with DNA samples derived from 30 individuals to test the availability of primers and amplification conditions. To assess the mutation frequency of potential polymorphisms, 50 DNA samples were randomly selected for PCR amplifications and then the genotypes of these indel sites were determined by 3.5% agarose gel electrophoresis, as described previously. Specifically, touch-down polymerase chain reaction (TD-PCR) was performed for fragments amplification. The 12.5 μL of a total PCR reaction mixture contained 1 μL of genomic DNA (20 ng/μL), 4 μL ddH_2_O, 0.5 μL of each primer (10 μM), and 6.5 μL 2 × Taq Master mix (including 200 μM dNTPs, 0.625 units of Taq DNA polymerase (Tsingke, Shanghai, China), and 1.5 mM MgCl_2_). The PCR reaction was performed as follows: initially, denature at 95 °C for 5 min and then 18 cycles of 30 s at 94 °C, 30 s at 65 °C for annealing, with a decrease of 1 °C per cycle, and 72 °C at 20 s for extending, then another 25 cycles of 30 s at 94 °C, 30 s at 50 °C, and 20 s at 72 °C; finally, maintain at 72 °C for 10 min.

Genotypes of each mutation were distinguished according to the results of agarose gel electrophoresis. The indel polymorphism sites have three genotypes, insertion/insertion (II), insertion/deletion (ID), and deletion/deletion (DD), of which the II genotype shows one band, ID genotype shows two bands, and DD genotype shows one band. When the amplification products of each pair of primers presented different genotypes, the products were sequenced. Ultimately, all loci from individuals tested in this study were genotyped based on the amplified fragments.

### 2.5. Statistical Analyses

The genotype frequency and the allele frequency of the bovine *ITGβ5* gene were directly calculated based on the genotyping results. Additionally, based on the GDIcall Online Calculator (http://www.msrcall.com/Gdicall.aspx) (accessed on 27 May 2021), the Hardy–Weinberg equilibrium (HWE) and polymorphism information content (PIC) were calculated. Analysis of variance (ANOVA) was undertaken using SPSS (v24.0; IBM Corp., Armonk, NY, USA) to evaluate the associations between gene polymorphisms and ovarian traits. The adjusted linear model was Y_ij_ = μ + G_i_ + E_ij_, where Y_ij_ was the trait measured for each animal, μ was the overall mean, G_i_ was the type of the i-th genotype, and E_ij_ was the random error. Moreover, the results were considered statistically significant at a *p* value < 0.05.

## 3. Results

### 3.1. Identification of 13-bp Indel Polymorphism of the Bovine ITGβ5 Gene

**Figure 1:**
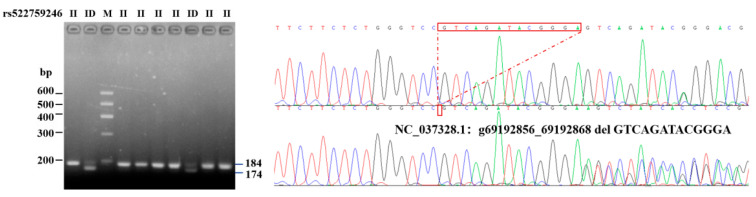
Electrophoretogram and sequence diagram of P1-D_13-bp_ of *ITGβ5* gene.

According to the genome sequencing results, the six potential variant loci were aligned to the genome sequences and its downstream area available in the NCBI database for reference (GenBank ID: 282564, NC_037328.1: 69192856-69312146).Among the six potential variant loci, rs522759246 (g. 69192856–69192868 del GTCAGATACGGGA), which is located downstream 4000 bp of the bovine *ITGβ5* gene, was polymorphic. Each indel polymorphism presents three different variations: homozygous insertion/insertion (II), homozygous deletion/deletion (DD), and heterozygous insertion/deletion (ID) (Figure 1). Depending on the types and location of the mutation, it was also named as primers1-deletion-13 bp (P1-D_13-bp_).

### 3.2. Genetic Diversity of the Novel 13-bp Indel Locus within ITGβ5 in the Population

After genotyping the amplified products of the P1-D_13-bp_ locus from 696 ovarian DNA samples, the genotype and allele frequencies were calculated. As shown in Table 2, the frequency of DD genotype was 0.072 and the allele D frequency was 0.152. Population parameters were also derived. The effective allele number (Ne) was 1.346. Meanwhile, the value of the polymorphic information content (PIC) was 0.224, indicating that the investigated population belonged to low-degree PIC (0.00 < PIC < 0.25) in the locus P1-D_13-bp_ [27]. The expected heterozygosity (He) was 0.257, which was higher than the observed heterozygosity value (0.160). Furthermore, the *p* value of the Hardy–Weinberg equilibrium (HWE) was 1.200E-23, suggesting a deviation from the HWE in this population (*p* < 0.05) (Table 2).

### 3.3. Association Analysis between 13-bp Indel of ITGβ5 and Phenotypic Characteristics of the Bovine Ovary

Next, we performed correlation analysis between the genotypes of P1-D_13-bp_ and ovarian morphological traits. Before that, all tested individuals were estimated by types of corpora lutea, follicle and corpus albicans, and 90% of female cows which for correlation analysis are not in oestrus. As shown in Table 3, these genotypes were not significantly associated with ovarian length, ovarian height, or ovarian weight. However, it is worth noting that the P1-D_13-bp_ was significantly associated with ovarian width (*p* = 0.015).

### 3.4. Relevance Analysis of the 13-bp Indel of ITGβ5 with Corpus Luteum Traits, Mature Follicle Follicles Traits and Corpus Albican Traits of the Bovine Ovary

During oogenesis, the oocytes are contained in the follicles, and the follicles develop in the corpora lutea after oocyte release. Accordingly, this study further investigated the relationship between the *ITGβ5* gene polymorphism and luteal traits, mature follicle traits and corpus albican traits. The P1-D_13-bp_ locus was also significantly correlated with corpus luteum diameter (*p* = 0.005). Moreover, the P1-D_13-bp_ locus tended to be associated with associated with the number of corpora lutea (*p* = 0.075) although no statistical significance was observed (Table 4).

Given that follicle growth and size, and thus the ovary dimensions, depend on the estrous cycle phases, associations between *ITGβ5* polymorphisms and ovarian traits were also analyzed in nearly 10% of oestrous individuals, for which the results of the ANOVA analysis were very similar compared to the non-oestrous group (Supplemental Table S1).

## 4. Discussion

The endometrial and corpus luteal transcriptomes contribute significantly to phenotypic fertility differences and the ovary (ovarian localization, corpus luteum, ovarian size, and weight) is important for evaluation of possible ovarian embryo production in vitro [10,28]. Our association analysis between mutations in *ITGβ5* and ovarian traits, as well as luteal traits, showed that P1-D_13-bp_ of the *ITGβ5* gene was significantly associated with the ovarian width and corpus luteum diameter of Holstein cows. It can be inferred from the results that genetic variation in the *ITGβ5* gene is likely to have important effects on ovarian phenotype and related traits, as well as luteal traits. Consistent with previous studies, integrin β5 binds to the ECM and mediates several physiological processes of cellular activity and apoptosis, and, therefore, is important for bovine ovarian development [15]. Moreover, it has been well documented that ITGβ5 is critical in the selection of ovarian follicles due to its control of cell proliferation and apoptosis, and its expression is upregulated in large follicles relative to small ones [16,17].

The mechanisms of action of integrins might explain the reason why the mutation in *ITGβ5* can affect the fertility of bovines. Integrins control cell attachment to the ECM and are involved in cell–cell and cell–ECM interactions [13,14]. When ECM ligands bind to integrins, several signaling proteins and adaptins are recruited to the integrin cytoplasmic domain, activating downstream signaling pathways [29,30]. It has been reported that integrins also exert significant effects on the survival, proliferation, and migration of cells [31]. Furthermore, fusion between the sperm and oocyte relies on intercellular and intercellular matrix interactions [32]. As we know, the endometrium promotes the development of the embryo through histotroph secretions and is also involved in the regulation of the estrous cycle. From another perspective, embryo implantation needs a process of adhesion between the embryo and uterine endometrium. Together, several studies indicate that ITGβ5 is highly conserved in different species and that integrins play multiple roles during fertilization, embryogenesis, and stages of implantation [33].

The data from our study showed that P1-D_13-bp_ was located at 4000 bp downstream of the bovine *ITGβ5* gene. Among all genetic variants in integrin genes, variants in the regulatory 3′ UTR region might have strong impacts on gene expression. The microRNAs (miRNAs), which might regulate integrins by binding to the target sites in 3′ UTR, thereby alter the expression of the *ITGβ5* gene. Although there are many challenges in the identification of biologically relevant miRNA targets, an increasing body of evidence suggests that alterations in miRNA target sequences can lead to diseases or changes in physiological state [34,35,36]. Therefore, it is tempting to speculate that the mutations in the 3′ UTR might affect the expression of the *ITGβ5* gene by affecting the binding of transcription factors and miRNAs. The tested locus did not conform to the Hardy–Weinberg equilibrium, a situation possibly due to the effect of selection, which needs further exploration.

## 5. Conclusions

In this study, an indel locus was identified within the bovine *ITGβ5* gene and was significantly correlated with bovine ovarian phenotypic trait and luteal trait. These results can be considered as preliminary in relation to fertility of bovines, whereas additional studies have to be undertaken to estimate the effect of *ITGβ5* gene of bovines’ fertility to verify that *ITGβ5* could be a new target gene for the application of MAS in breeding highly fertile bovines.

## Figures and Tables

**Table 1:** PCR primer sequences for *ITGβ5* amplification.

Locus	Rs Number	Primer Sequences (5′–3′)	Product Size (bp)	*Tm* (°C)	Region
P1-D_13-bp_	rs522759246	F1: GTTCCTGCTCAAGTCTCGGG R1: CTTCCACTCTCACCCCCAAC	187/174	60.39	Downstream 4000 bp
P2-D_12-bp_	rs450642714	F2: TGACTGCCTGCCAAATGTCC R2: TCTGCTCTCCCTCCTCCTTT	247/235	60.90	Downstream 4000 bp
P3-D_10-bp_	rs135754430	F3: CTGCTCAAGTCTCGGGGATT R3: TCTTCCACTCTCACCCCCAA	184/174	60.10	Downstream 4000 bp
P4-D_10-bp_	rs797103610	F4: CTGTCTCCCCTTCCACACAC R4: TCTCCTAGAAGGTCACCGCA	151/141	59.96	Downstream 2000 bp
P5-D_9-bp_	rs468306533	F5: TTTCTCCCGTGCGTGTATGT R5: CCCCTCTGATCTCCCCATCT	247/238	59.81	Downstream 1000 bp
P6-D_9-bp_	rs136097870	F6: CAGCACACAGAGGCAACAAC R6: AATCACCGCCAGCTTTGAAC	239/230	59.97	Downstream 1000 bp

Note: P indicates pairs of primers and the numbers represent the positional order of the detection.

**Table 2:** Polymorphism parameters of P1-D_13-bp_ of bovine *ITGβ5* gene.

**Sizes**	**Genotypic Frequencies**			**Allelic Frequencies**		**HWE *p* Values**		**Population Parameters**	
	**II**	**ID**	**DD**	**I**	**D**	1.2 × 10^−23^	**He**	**Ne**	**PIC**
696	0.768	0.160	0.072	0.848	0.152		0.257	1.346	0.224

Note: HWE, Hardy–Weinberg equilibrium; He, expected heterozygosity; Ne, effective allele numbers; PIC, Polymorphism information content.

**Table 3:** Relationships between P1-D_13-bp_ polymorphisms of *ITGβ5* and ovarian morphological traits.

Sizes	Traits (Units)	Observed Genotypes (Mean ± SE)			*p* Values
		II (n)	ID (n)	DD (n)	
611	Ovarian length (mm)	42.25 ± 0.41 (462)	41.78 ± 0.93 (106)	40.40 ± 1.47 (43)	0.409
611	Ovarian width (mm)	23.49 ^b^ ± 0.35 (462)	25.75 ^a^ ± 0.65 (106)	24.60 ^ab^ ± 1.13 (43)	0.015
610	Ovarian height (mm)	25.44 ± 0.35 (461)	24.95 ± 0.68 (106)	26.84 ± 1.05 (43)	0.368
611	Ovarian weight (g)	11.49 ± 0.24 (463)	11.06 ± 0.52 (105)	10.45 ± 0.73 (43)	0.371

Note: Values with different letters (a,b) within the same row differ significantly at *p* < 0.05. n indicates the number of individuals of the corresponding genotype. SE means one standard error of the mean. Ovarian morphological traits are presented as the Mean ± SE.

**Table 4:** Relationships between P1-D_13-bp_ of *ITGβ5* and corpus luteum traits, mature follicle and corpus albican traits of the bovine ovary.

Sizes	Traits (Units)	Observed Genotypes (Mean ± SE)			*p* Values
		II (n)	ID (n)	DD (n)	
528	Corpus luteum diameter (mm)	13.82 ^a^ ± 0.44 (400)	10.60 ^b^ ± 0.78 (95)	13.33 ^ab^ ± 1.55 (33)	0.005
531	Number of corpora lutea	1.55 ± 0.48 (402)	1.81 ± 0.13 (95)	1.68 ± 0.20 (34)	0.075
157	Number of mature follicles	1.26 ± 0.05 (105)	1.35 ± 0.11 (37)	1.27 ± 0.12 (15)	0.672
156	Mature follicle diameter (mm)	12.27 ± 0.44 (105)	12.43 ± 0.79 (36)	13.90 ± 1.07 (15)	0.426
123	Number of corpora albicantia	1.55 ± 0.12 (92)	1.22 ± 0.88 (23)	1.75 ± 0.49 (8)	0.315
122	Corpus albican diameter (mm)	4.76 ± 0.36 (91)	4.70 ± 0.68 (23)	5.91 ± 1.71 (8)	0.653

Note: Values with different letters (a,b) within the same row differ significantly at *p* < 0.05. n indicates the number of individuals of the corresponding genotype. All morphological traits are present as the Mean ± SE.

## Data Availability

The datasets presented in this study can be found in online repositories. The names of the repository/repositories and accession numbers can be found below: https://www.ncbi.nlm.nih.gov/genbank/ (accessed on 27 May 2021), GenBank ID: 282564, NC_037328.1: 69192856-69312146.

## Supplementary Materials

The following are available online at https://www.mdpi.com/article/10.3390/ani11061579/s1, Table S1: Relationships between P1-D13-bp of ITGβ5 and ovarian related traits of the bovine ovary in oestrum.
