# Supplementary material for: Fertility-Associated Polymorphism within Bovine ITGβ5 and Its Significant Correlations with Ovarian and Luteal Traits
Source: Animals (Basel). 2021 May 28;11(6):1579. doi: 10.3390/ani11061579 (PMC8228251; doi:10.3390/ani11061579)
Supplement: Supplementary file 1 [file animals-11-01579-s001.zip › animals-1179266-supplementary.pdf]

**Table S1.** Relationships between P1-$D_{13\text{-bp}}$ of *ITGβ5* and ovarian related traits of the bovine ovary in oestrum.

| Sizes | Traits (units) | Observed Genotypes (Mean ± SE) | | | *P* Values |
|---|---|---|---|---|---|
| | | II (n) | ID (n) | DD (n) | |
| 80 | Ovarian length (mm) | 38.91 ± 0.98 (68) | 41.00 ± 2.30 (5) | 37.57 ± 4.40 (7) | 0.781 |
| 80 | Ovarian width (mm) | 19.88 ± 0.69 (68) | 24.60 ± 1.36 (5) | 18.43 ± 2.05 (7) | 0.145 |
| 80 | Ovarian height (mm) | 22.28 ± 0.75 (68) | 24.00 ± 2.41 (5) | 23.71 ± 2.08 (7) | 0.715 |
| 78 | Ovarian weight (g) | 8.52 ± 0.39 (66) | 9.44 ± 1.35 (5) | 8.25 ± 1.26 (7) | 0.794 |
| 23 | Number of mature follicles | 1.22 ± 0.10 (18) | 1.20 ± 0.20 (5) | 1.22 ± 0.09 (23) | 0.920 |
| 23 | Mature follicle diameter (mm) | 13.22 ± 1.20 (18) | 10.40 ± 1.75 (5) | 12.61 ± 1.02 (23) | 0.266 |
| 14 | Number of corpora albicantia | 1.27 ± 0.14 (11) | 2.00 (1) | 1.50 ± 0.50 (2) | 0.369 |
| 14 | Corpus albican diameter (mm) | 5.25 ± 0.73 (12) | 5.00 (1) | 15.00 (1) | - |

Note: Values with different letters (a, b) within the same row differ significantly at $p < 0.05$. n indicates the number of individuals of the corresponding genotype. All morphological traits are present as the Mean ± SE.
